# Prevalence, predictors of low birth weight and its association with maternal iron status using serum ferritin concentration in rural Eastern Ethiopia: a prospective cohort study

**DOI:** 10.1186/s40795-022-00561-4

**Published:** 2022-07-26

**Authors:** Meseret Belete Fite, Abera Kenay Tura, Tesfaye Assebe Yadeta, Lemessa Oljira, Kedir Teji Roba

**Affiliations:** 1 Department of Public Health, Institute of Health Sciences, Wollega University, Nekemte, Ethiopia; 2 School of Nursing and Midwifery, College of Health and Medical Sciences, Haramaya University, Harar, Ethiopia; 3 Department of Obstetrics and Gynaecology, University Medical Centre Groningen, University of Groningen, Groningen, the Netherlands; 4 School of Public Health, College of Health and Medical Sciences, Haramaya University, Harar, Ethiopia

**Keywords:** Iron, Iron deficiency anemia, Anemia, Birth, Wight, Birth cohort

## Abstract

**Introduction:**

Low birth weight (LBW) is one of the major predictors of perinatal survival, infant morbidity, and mortality, as well as the risk of developmental disabilities and illnesses in future lives. The effect of the nutritional status of pregnant women on birth outcomes is becoming a common research agenda, but evidence on the level of low birth weight (LBW) and its association with prenatal iron status in Ethiopia, particularly among rural residents, is limited. Thus, this study aimed to assess the prevalence, predictors of LBW, and its association with maternal iron status using serum ferritin concentration in Haramaya district, eastern Ethiopia, 2021.

**Methods:**

A community-based prospective cohort study design was conducted. Of a total of 427 eligible pregnant women followed until birth, 412 (96.48%) were included in the final analysis. Iron status was determined using serum ferritin (SF) concentration from venous blood collected aseptically from the ante-cubital veins analyzed on a fully automated Cobas e411 (German, Japan Cobas 4000 analyzer series) immunoassay analyzer. Iron deficiency(ID) and iron deficiency anemia (IDA) were classified as having SF less than 15 μg/L and SF less than 15 μg/L and Hb level of < 11.0 g/dl during the first or third trimester or < 10.5 g/dl during the second trimester as well, respectively. Birthweight was measured within 72 h of birth and < 2500 g was considered LBW. Birthweight was measured within 72 h of birth and < 2500 g was considered as LBW. A Poisson regression model with robust variance estimation was used to investigate the factors associated with LBW and the association between maternal iron status and LBW. An adjusted prevalence ratio with a 95% confidence interval was reported to show an association using a *p*-value < 0.05.

**Results:**

About 20.2% (95% CI: 16%-24%) of neonates were born with LBW. The prevalence of LBW was 5.04 (95% CI = 2.78–9.14) times higher among women who were iron deficient during pregnancy compared to those who were normal. The neonates of women who were iron deficient during pregnancy had lower birth weight (aPR=5.04; 95% CI = 2.78–9.14) than the neonates of women who were normal. Prevalence of LBW was higher among mothers who were undernourished (MUAC < 23cm) (aPR = 1.92; 95% CI= 1.33–2.27), stunted (height <145cm) (aPR=1.54; 95% CI=1.04–2.27) and among female neonates (aPR=3.70; 95% CI= 2.28–6.00). However, women who were supplemented with iron and folic acid (IFAS) during pregnancy had a 45% decreased chance of delivering low birth weight (aPR= 0.55; 95% CI=0.36–0.84).

**Conclusion:**

We found that LBW is of public health significance in this predominantly rural setting. ID during pregnancy is found to have a negative effect on birth weight. IFA supplementation, the maternal under-nutrition, height, and sex of neonates were identified as predictors of low weight at birth. To improve maternal nutritional status, health interventions must address targeted strategies promoting desirable food behavior and nutritional practices. These include; promoting the consumption of diversified and rich iron food to improve the maternal nutritional status. A continued effort is needed in enhancing universal access and compliance with IFA supplementation to improve maternal health. Intervention strategies that are complementary and comprehensive across the vulnerable periods for women during pregnancy and their neonates that are based on a life-cycle approach are suggested.

## Introduction

Pregnancy is associated with considerable changes in the physiological, anatomical, and biochemical attributes in women that affect the fetus’s developmental stages and, subsequently, impact the neonates’ birth weight and other related birth outcomes, such as gestation age at birth, stillbirth, and neonatal mortality [1]. Moreover,​macro-and micro-nutrient requirements rise during pregnancy to provide for physiological and metabolic changes and fetal development [1]. World Health Organization (WHO) defined LBW as the weight of a neonate below 2500 g at birth, irrespective of the gestational age of the neonate [2, 3]. Worldwide, low birth weight (LBW) remains an important public health concern [2]. Evidence showed that globally 15 to 20% of all live births each year are of LBW, and of all neonatal deaths, over 80% occurred in LBW newborns [4, 5].

The prevalence of LBW differs across regions in Ethiopia. Several studies tried to assess the prevalence of LBW have witnessed the prevalence of LBW ranging from 8.6 to 28.3% [6–9]. However, most of the studies were facility-based. Also, a community-based prospective study in rural Sidama, southern Ethiopia reported 16.5% [10]. Moreover, 2011 [11] and 2016 [12] Ethiopian demographic health surveys (EDHS) documented an 8% and 16% prevalence of LBW based on mothers' reporting, needless the estimate might not be trustworthy because of the reporting mistakes and recall bias. Moreover, a recent systematic review and meta-analysis reported a 17.3% prevalence of LBW [13]. The WHO has identified LBW as public health priority and targets a 30% reduction in LBW globally between 2012 and 2025 [14]. Nevertheless, the DHS reports showed an increasing trend in the prevalence of LBW in Ethiopia [11, 12].

Women in low-income countries often enter pregnancy with micronutrient deficiencies. They are exacerbated in pregnancy due to the increased demands of the developing fetus, leading to potentially adverse effects on the mother and babies [15]. Iron deficiency is a common complication that increases the risk of adverse pregnancy outcomes, such as low birth weight, preterm birth, perinatal and infant mortality, postpartum hemorrhage, and spontaneous abortion [16, 17]. This implies that pregnant women face a double burden of health risk because they have increased nutrient demand to ensure their own growth/development, in addition to that of their growing fetuses.

Studies endeavored to determine the effect of prenatal iron deficiency on birthweight concluded ambiguously [18–24]. Evidence from the literature concludes that maternal iron deficiency negatively affects birth weight [22–26]. However, to our knowledge, this is the first report to examine neonates' birth weight and its association with maternal iron status using SF concentration in Ethiopia, particularly in the rural community-based setup. Therefore, this study aimed to assess the prevalence, predictors of LBW, and its association with maternal iron status using SF concentration in Haramaya district, eastern Ethiopia.

## Methods and materials

### Study settings

The study was embedded into the Haramaya Health Demographic Surveillance and Health Research Centre (HDS-HRC), which was established in 2018. The HDS-HRC covers 12 rural kebeles (the lowest administrative unit in Ethiopia) out of 33 kebeles found in the district located approximately 500 KM away from the capital city, Addis Ababa. From 5252 pregnant women in the district during the study period, 2306 were under follow-up of the HDS-HRC [27, 28].

### Study design and population

**Fig. 1:**
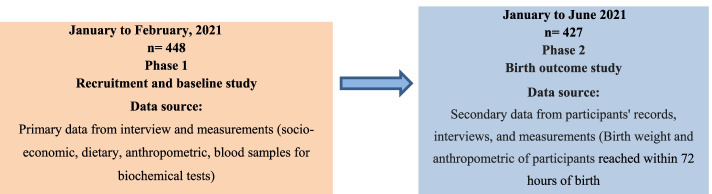
Study flow shows date and duration, study design, and data collected

A community-based prospective cohort study was conducted. All pregnant women whose serum ferritin and hemoglobin concentrations were determined at their second or third trimester and who later gave singleton live births were eligible for the study. Neonates reached later on 72 h of birth were excluded. We also excluded pregnant women whose exposure was determined in the first trimester, as the fetal weight gain in the first trimester is known to be minimal. This study is a longitudinal study that obtained birth outcome information of pregnant women, the exposure of LBW was determined from Charan and Biswas's study [29], with the input of 95% confidence level, 5% marginal error, 80% power, and 1:1 ratio between exposed and non-exposed subjects. The expected prevalence of LBW in exposed and non-exposed subjects was taken from prior studies [30]. As a result, 432 sample sizes were computed. Nevertheless, by adding a 10% non-response rate 475 participants were included. After constructing a sampling frame from the HDS-HRC database, simple random sampling was applied to the eight randomly selected kebeles and then the eligible women were selected using the computer-generated lottery method. Nevertheless, for this specific paper, 427 pregnant women who were in their second or third trimester during the baseline survey were considered. Figure 1 displays a detailed description a flow diagram for study into two phases.

### Recruitment

As described in the previous papers [31, 32], from 475 calculated sample sizes, 448 pregnant women were included in the baseline survey. For this specific paper, 427 whose serum ferritin and hemoglobin concentrations were determined at their second or third trimester during the baseline survey were considered. Of 427 followed pregnant women, birth weight was taken from 412 neonates, as lost to follow-up [2], multiple births [3] and early neonatal death [2], birth weight measurement taken after 72 h of birth [3], and fetal loss [5] were excluded. We recruited participants selected from the HDS-HRC database voluntarily from the community during home-to-home visits. We obtained days in which antenatal care services were not held for pregnant women in the nearby health facilities. On these dates, researchers visited the randomly selected kebeles and eligible pregnant women required for recruitment. Although special attention was given to involving all eligible pregnant women for participation, some of them did not volunteer to participate in the cohort. We made announcements using health extension workers to invite pregnant women to select kebeles in the district.

### Baseline study

Upon recruitment and signing of informed consent, we assessed socio-economic characteristics, dietary practices, anthropometric measurements, and collected blood samples for hematology and biochemical analysis. We carried out the face-to-face interviews to collect knowledge, attitudes, and obstetric and other pregnancy-related data on house-to-house visits. The two-day training was given by research experts to train data collectors, laboratory professionals, and supervisors before the pre-test on each data collection tool. A day field pre-testing session followed the training in a nearby rural district. These trained data collectors carried out all data collection for this study at the randomly selected kebeles in the HDS-HRC.

### Follow-up study

After the baseline study, participants' telephone numbers and addresses were collected and entered into a book, and we entered the expected date of delivery for each participant. The occurrence of births in the cohort was promptly found and informed by pre-established local community health promoters which the country labeled as ''Women Developmental Army (WDA)" and health extension workers (HEW). We used a structured questionnaire to gather data on the birth experiences of each participant. Birth outcomes such as gestational age at birth, birth weight, birth delivery method, neonatal mortality, stillbirth, abortions, and postpartum morbidity of mothers were obtained. Among all the birth outcomes data, birth weight was used as the outcome variable in this study. Birth weight was gauged within 72 h of birth by trained and well-experienced data collectors who were employed by HDS-HRC. Among all the birth outcomes data, birth weight was used as the outcome variable in this study.

### Data collection methods

As described in the previous papers [31, 32], in the baseline survey, data were collected through face-to-face interviews by trained research assistants using a standard pretested questionnaire (food frequency questionnaire) translated to the local language. The questionnaire contains data on socio-economic, obstetric, maternal perception, food consumption, dietary diversity, knowledge, attitude, and practices of pregnant women. Dietary diversity was assessed using the validated Food Frequency Questionnaire (FFQ), containing 27 commonly the list of food items consumed [33–37]. In addition, Mid-Upper Arm Circumference (MUAC) and maternal height measurements were taken. The questionnaire was initially prepared in English language and translated to the local language (Afan Oromo) by individuals with good command of both languages. It was also pre-tested on 10% of the sample in Kersa District before actual implementation.

A 5 ml venous blood was aseptically drawn from the antecubital veins and aliquoted into plain test tubes without anticoagulants. The blood samples were centrifuged, followed by separation of serum, stored frozen at -80 °C, and analyzed at the national chemistry laboratory in Ethiopian Public Health Institute (EPHI). We measured SF and serum high-sensitive C-reactive protein (hsCRP). SF was analyzed on a fully automated Cobas e411 (German, Japan Cobas 4000 analyzer series) immunoassay analyzer by the electro-chemiluminescence (ECL) method using commercial kits supplied by Roche Company, Germany at National Clinical Chemistry Reference Laboratory, EPHI. Whereas, highly sensitive C-protein reactive (hsCRP) was analyzed by Roche/Hitachi Cobas 6000 (c501): (German, Japan Cobas 6000 series of Roche) fully automated clinical chemistry analyzer [38]. The tests were performed by trained and experienced medical laboratory technologists.

Two levels of quality control (QC) samples were performed at least once every 24 h when the test is in use, once per reagent kit, and following each calibration to evaluate the functionality of the instrument and reagent, and the results of QC were evaluated using the Levey–Jennings chart (Wesgard rules). The calibration method has been standardized against the WHO International Standard NIBSC code: 03/178, 1st International Standard (IS) NIBSC (National Institute for Biological Standards and Control) "Reagent for Ferritin (human liver)" 80/602, and Reference preparation of the IRMM (Institute for Reference Materials and Measurements) BCR470/CRM470 (RPPHS-Reference Preparation for Proteins in Human Serum) for serum ferritin and serum hsCRP respectively. Calibration was performed as per the standard operating procedures (SOPs).The high ferritin cut-off point (SF < 15 μg/L) recommended by WHO for developing countries was used to define ID to compensate for the effect of infection, which can lead to elevation of the level of ferritin. Serum ID, moderate iron depletion, and ID were termed as SF less than 15 μg/L, 15–30 μg/L, and > 30 μg/L [39]. Serum CRP levels higher than 5 mg/L were termed as high CRP [33]. Anemia was defined as a Hb level of < 11.0 g/dl during the first or third trimester or < 10.5 g/dl during the second trimester [39]. Hemoglobin concentration was measured at each study site by well-trained medical technologists from capillary blood using a portable HemoCue Hb 301®, which is a gold standard for fieldwork. Hemoglobin values were adjusted for altitude as per the Center for Disease Prevention and Control (CDC) recommendation [40]. The significance of babies was computed to the nearest 100 g using calibrated Docbel BRAUNH scale). A LBW was defined as a live birth baby born with a birth weight of < 2500 g, and control was defined as a live birth baby born with a birth weight of ≥ 2500 g [41].

To estimate the economic level of families, a wealth index was employed. The wealth dispersion was generated by applying principal component analysis (PCA). The index was calculated based on the ownership of latrine, agricultural land and size, selected household asset, a quantity of livestock, and source of water for drinking containing 41 household variables. As described in the previous paper [31] nutritional knowledge and attitude towards consumption of an iron-rich diet were evaluated with questions using the Likert scale using PCA and the factor scores were totaled and classified into terciles. Women's autonomy was evaluated by seven validated questions which were adopted from the Ethiopian demographic health survey [12]. For each question, the response was coded as "one" when the decision is made by the woman alone or jointly with her husband, or "zero" otherwise.

### Data quality assurance

The detailed methods of data quality assurances used are described in previous papers (3,321), in the baseline survey. Quality assurance during laboratory analysis was monitored in the National Reference Laboratory for Clinical Chemistry at the Ethiopian Public Health Institute (EPHI). The EPHI laboratory is accredited by the Ethiopian National Accreditation Office (ENAO) to conduct tests under ISO 15189:2012, Quality and Competence Medical Laboratory Requirements (accreditation no. M 0025) by well-trained and experienced laboratory professionals and standard operating procedures were strictly followed for respective parameters.

### Data processing and analysis

Data were double entered using EPiData version 3.1 software. Data were cleaned, coded, and checked for missing and outliers, for further analysis and exported to STATA version 14 (College Station, Texas 77,845 USA) statistical software. The outcome variable was dichotomized as LBW (= 1) if newborns were born with a birth weight of < 2500 g or normal (= 0), otherwise. Thus, the Poisson regression analysis model with a robust variance estimate was fitted to identify predictors of LBW. Bivariate analysis and multivariable analyses were done to identify the association between independent variables and LBW. The backward regression was fitted with selected socio-economic and fertility-related variables. The goodness of fit was checked by Hosmer–Lemeshow statistic and omnibus tests. Possible interactions between covariates were tested. Akaike's information criterion (AIC) and Bayesian information criterion (BIC) were used to test for model fitness. All variables with *p* < 0.25 in the binary analyses were included in the multivariable analysis after checking for multi-collinearity using variance inflation factors. Adjustments were done for independent variables (age, Mid-Upper Arm Circumference, maternal height, sex of neonates, hemoglobin level, C-reactive protein) for assessing iron status and birth weight. The direction and strength of statistical association were measured using adjusted prevalence ratio (aPR) along with their corresponding 95% confidence interval (CI). An adjusted prevalence ratio (aRR with a 95% confidence interval was reported to show an association at a *p*-value < 0.05.

To estimate the economic level of families, a wealth index was employed. The wealth dispersion was generated by applying principal component analysis (PCA). The index was calculated based on the ownership of the latrine, agricultural land and size, selected household assets, the quantity of livestock, and source of water for drinking containing 41 household variables. As described in the previous paper [31], nutritional knowledge and attitude towards consumption of an iron-rich diet were evaluated with questions using the Likert scale using PCA, and the factor scores were totaled and classified into tertiles. Women's autonomy was evaluated by seven validated questions which were adopted from the Ethiopian demographic health survey [37]. For each question, the response was coded as "one" when a decision is made by the woman alone or jointly with her husband or "zero" otherwise.

### Ethical consideration

All methods of this study were carried out under the Declaration of Helsinki's ethical principle for medical research involving human subjects [12]. Ethical approval was obtained from the Institutional Health Research Ethics Review Committee (ref no: IHRERC/266/2020) College of Health and Medical Sciences, Haramaya University before the commencement of the study. Written informed consent was obtained from each participant or legally authorized representatives for those below 16 years of age. The interview was conducted in private and confidentiality of the participants' information was maintained.

## Results

### Socio-demographic characteristics

Of a total of 427 followed eligible pregnant women, 412 (96.48%) were included in the final analysis. The retained and excluded participants did not significantly vary by wealth index, maternal age, educational status, nutritional status, iron status, anemia status, CRP and status, and dietary diversity (*p* > 0.05). Of women included in the study 107(25.97%), 187(47.82%), and 193(46.84%) were stunted, undernourished, and iron deficient respectively. The majority of the respondents could not read or write (73.79%). Only 20.63% of the respondents were in the richest quintiles, Table 1

**Table 1:** Comparability of retained and excluded subjects based on key socio-demographic and nutritional factors in Haramaya district, Eastern Ethiopia, 2021

Variable	Excluded subject (*n* = 15)	Included subjects (*n* = 412)	*P*- Value
	**n (%)**	**n (%)**	
Age (years)			0.190
< 18	21(5.10)	2(13.33)	
> 18	391(94.90)	13(86.67)	
The educational level of the woman			0.900
Can’t read or write	304(73.79)	11(3.33)	
Read or write	24(5.83)	1( 6.67)	
Formal education	84(20.39)	3(20.00)	
Wealth Index (Quintile)			0.069
Poorest	84(20.39)	2(13.33)	
Poor	81(19.66)	5(33.33)	
Middle	83(20.15)	2(13.33)	
Rich	79(19.17)	5(33.33)	
Richest	85(20.63)	1(6.67)	
Maternal height (cm)			0.432
Normal (≥ 145 cm)	305(74.03)	12(80.00)	
Stunted(< 145 cm)	107(25.97)	3(20.00)	
Nutritional status (MUAC)			0.571
Normal (MUAC < 23 cm)	215(52.18)	8(53.33)	
Under-nutrition (MUAC ≥ 23 cm)	187(47.82)	7(46.67)	
Iron status (SF)^b^			0.401
Normal (SF ≥ 15)	219(53.16)	9(60.00)	
Iron deficient(SF < 15)	193(46.84)	6(40.00)	
Anemic status (hemoglobin level)^a^			0.592
Normal	221(53.64)	8(53.33)	
Anemic	191(46.36)	7(46.67)	
C-reactive protein (CRP)			0.122
Negative (CRP < 5)	320(77.67)	9(60.00)	
Positive (CRP ≥ 5)	92(22.33)	6(40.00)	
Dietary diversity			0.569
Low	121(70.63)	3(80.00)	
High	291(29.37)	12(20.00)	

Percentages refer to the proportion of participants who were followed for birth outcomes and are calculated from the column total

A fisher's exact test was used to calculate the *P* value

^a^Anemia was defined as a Hb level of < 11.0 g/dl during the first or third trimester or < 10.5 g/dl during the second trimester

^b^Iron deficiency and iron deficiency anemia were classified as having SF less than 15 μg/L and SF less than 15 μg/L and Hb level of < 11.0 g/dl during the first or third trimester or < 10.5 g/dl

### Prevalence and general determinants of low birth weight

The mean neonates' birth weight was 2916.75 (± 546.55) grams. The mean male and female neonates’ birth weight was 2996.244 (± 486.60) and 2831.658 ((± 593.70) grams respectively. About one-fifth (20.2%) of neonates (95% CI: 16%-24%) were born with LBW. An independent t-test was used to identify if there is a significant difference in the birth weight of the neonates among the sex group of the newborn. Hence, we conclude that there is a significant difference in birth weight among the sex groups (*p*-value < 0.05). In the bi-variable analysis supplementations of IFA, occupation of the woman, sex of neonates, fertility desire, time to health facility, maternal MUAC, and maternal height were found to be a candidate for multivariable analysis at p < 0.25. The prevalence of LBW was higher among mothers who were undernourished (MUAC < 23 cm) (aPR= 1.92; 95% CI=1.33–2.27), stunted (height <145 cm) (aPR=1.54; 95% CI = 1.04–2.27) and among female neonates (aPR=3.70; 95% CI=2.28–2.6.36). However, women who were supplied with iron and folic acid during pregnancy had a 45% decreased chance of delivering LBW neonates (aPR= 0.55; 95% CI = 0.36–0.84), Table 2

**Table 2:** General determinants of low birth weight in Haramaya District, Eastern Ethiopia, 2021 (*n* = 412)

Variables	Birth weight		cPR (95% CI)	aPR (95% CI)	*P*-value
	Low (*n* = 83)	Normal (*n* = 329)			
Supplementations of IFA					
No	62(74.70)	194(58.97)	1	1	
Yes	21(25.30)	135(41.03)	0.56(0.35, 0.87)	0.55( 0.36, 0.84)	0.005
Occupation of the woman					
Housewives	78( 93.98)	321(97.57)	1	1	
Merchant	5( 6.02)	8( 2.43)	1.97(0.96, 4.03)	1.70(0.86, 3.38)	0.129
Sex of neonates					
Males	*18(21.69)*	195(59.27)	1	1	
Females	65(78.31)	134(40.73)	3.86(2.38, 6.28)	3.70(2.28, 6.00)	< 0.001**
Maternal nutritional status (MUAC)					
Normal (MUAC ≥ 23)	33(39.76)	182(55.32)	1	1	
Under-nutrition (MUAC < 23)	50(60.24)	147( 44.68)	1.65(1.12, 2.46)	1.92( 1.33, 2.76)	0.001*
Fertility desire					
No	31(37.35)	101(30.70)	1	1	
Yes	52(62.65)	228(69.30)	0.79(0.53, 1.17)	0.78(0.54, 1.15)	0.207
Maternal height in cm					
> 145 cm	56 (67.47)	249(75.68)	1	1	
< 145 cm	27(32.53)	80(24.32)	1.37(0.92, 2.06)	1.54(1.04, 2.27)	0.029*
Time to the health facility					
< 40 min	63(75.90)	270(82.07)	1	1	
> 40 min	20(24.10)	59(17.93)	1.34(0.86, 2.08)	1.40(0.93, 2.09)	0.103

*cPR* crude Prevalence Ratio, *aPR* adjusted Prevalence Ratio

^**^Statistically significant at *p*-value < 0.001

^*^statistically significant at *p*-value < 0.05

### Associations between neonates’ birth weight and maternal iron status

In the multivariable model adjustments was done for potential confounders for (age, MUAC, maternal height, sex of neonates and hemoglobin level, and C-reactive protein), which were statistically associated with low birth weight in the bivariate analysis. The adjusted modes revealed significant associations between prenatal iron status and birth weight. The prevalence of low birth weight was 5.04 (95% CI = 2.78–9.14) times higher among women who were iron deficient during pregnancy compared to those who were normal, Table 3.

**Table 3:** Multivariable Poisson regression analysis model of the association between neonates’ birth weight and maternal iron status in Haramaya district, eastern Ethiopia, 2021 (*n* = 412)

Variables	Birth Weight		Prevalence ratio	95% CI
	Low (*n* = 83)	Normal (*n* = 329)		
Prenatal iron status				
**Crude model**				
Normal	11(13.25)	182(55.32)	1	1
Iron deficient	72(86.75)	147(44.68)	5.77	3.149, 10.563
**Adjusted mode**^a^				
Normal	11(13.25)	182 (55.32)	1	1
Iron deficient	72(86.75)	147(44.68)	5.04	2.778, 9.143^b^

^a^Adjusted for age, Mid-Upper Arm Circumference, maternal height, sex of neonates and hemoglobin level, C-reactive protein

^b^Statistically significant

## Discussion

This study aimed to assess the prevalence, predictors of low birth weight, and its association with maternal iron status using serum ferritin concentration in Haramaya district, eastern Ethiopia. The prevalence of LBW was 20.2% (95% CI: 16–24%). Factors associated with LBW were supplementations of IFA, maternal under-nutrition, stunting, and sex of neonates. We found that there is an increased risk of low birth weight in neonates born to iron-deficient mothers.

Low birth weight is considered a public health concern when it exceeds 15% [42]. There is significant variation in the prevalence of LBW across regions and within countries; nevertheless, the great majority of LBW occurs in low-and middle-income countries and especially in the most vulnerable populations [43, 44]. The prevalence of low birth weight in the current study is comparatively higher than the findings of studies conducted in rural Sidama, southern Ethiopia [13], but lower than the reports of studies carried out in the Southern Gonder zone, Northwestern Ethiopia [36] and in Public Hospitals of Harari Regional State, Eastern Ethiopia [45]. The possible variations might be the difference in the sociocultural and study design used that most of the studies were conducted in the health facilities. The current research witnessed a relatively higher prevalence probably because it was carried out in a rural area where the burden of the problem is expected to be high.

Various investigations have shown a positive association between IFA supplementation and fetus development [46, 47]. In this study, we revealed that IFA supplementation during pregnancy is related to increased birth weight. Mothers who received IFA supplementation decreased the chance of delivering LBW. This finding is in line with a study carried out in Ghana that revealed supplementation of IFA in pregnancy was found to have a protective effect against LBW [48]. Moreover, this result is also in agreement with other related previous studies conducted in Ethiopia [49, 50]. Supplementation of IFA in pregnancy raises hemoglobin levels and prevents women from becoming anemic and subsequently reduced the risk of giving LBW neonates because the needed quantity could not be supplied from dietary consumption during this period [51]. Although the mechanism of IFA supplementation on birth weight is known, this might be due to the reality that taking IFA supplementation increases the appetite of the mother and leads to improving the general nutritional status of mothers which consecutively lessens the chance of having the LBW neonate and the growing fetus shares nutrients from the mother for its intrauterine development. We observed maternal MUAC was strongly correlated with birth weight both in the Poisson regression with robust variance estimate models. Our finding is comparably in agreement with studies conducted in Ethiopia and abroad [52–54], reported as well. As pregnant women are often registered into supplementary and therapeutic feeding programs grounded on their MUAC in Ethiopia, the current study could have underestimated the association. The fact that improved nutritional value of food for optimal maternal health has a better pregnancy outcome and offspring health, food approach interventions must address targeted strategies where nutrition anemia is prevalent in this study setup.

In this study, maternal height was a significant predictor of LBW. Our finding is in agreement with the reports of studies carried out in Cameroon [55], India [56], and Ethiopia [57, 58], where maternal stunting was a significant determinant of low birth weight. This is in line with the concept that in the developing world just about 12% of LBW can be explained by maternal short stature [57]. Even though the accurate mechanism of how maternal height affects newborns' weight at birth is not distinct, it could be due to the mixed indication of genetic and inter-generational consequences of malnutrition [58]. Another possible reason could be due to the concept that women who are short in height may also have a narrow pelvis, last with restricted intrauterine space that limits intrauterine fetal growth, and end up with LBW [59]. In the present study, the newborns of women who were iron deficient during pregnancy had on average 524.73 g lower birth weight than the newborns of women who were normal. Thus, male babies were observed heavier than females, and the risk of low birth weight was increased in female babies. Also, several studies carried out in Adds Ababa, Ethiopia [60], Metu, Southwest Ethiopia [61], and Sidama, Southern Ethiopia [10], witnessed as well. The result is in the ratification of the conclusion of a meta-analysis that on average male babies weigh 150 g heavier than females [62]. This could be due to biological and genetic factors [58].

The current study has witnessed a significant association between prenatal iron status and infants' birth weight. From our result, babies born to iron-deficient mothers were lighter than their counterparts. Our finding is in line with previous studies reporting the positive correlation between maternal serum ferritin and birth weight [22, 61, 63]. This is could be because IDA leads to change in norepinephrine, cortisol, and corticotrophin which results in oxidative stress to fetal growth leading to having an LBW newborn. However, some observational studies conducted in the developed and developing world reported no association between prenatal iron deficiency and birth weight [8, 23, 24]. However, almost all of the studies reported no association merely based on correlation or t-test analyses. Among the studies, only a few [38] applied multivariate analyses to control potential confounders. Accordingly, the overwhelming lack of association reported in the literature can be due to the effect of confounding factors as most of the studies did not adequately control potential extraneous variables. Moreover, it is essential to interpret the result in consideration of the reality that the sample size of the study was only calculated for the predictors of birth weight. Therefore, in the evaluation of the association between prenatal birth weights, the power of the study could have been compromised. Consequently, in this study setup, further research with optimal sample size is needed.

The important strength of this study was that data were collected prospectively with multiple variables. Additionally, serum ferritin concentrations biomarkers have been used to measure iron status. Especially to make a decision on the co-occurrence of inflammation in the interpretation of the finding of iron status, an analysis of conceptually important confounder (CRP) was carried out and was used for the adjustments of serum ferritin concentration in the present study. The study limitations should be considered when interpreting this result. The reality that prenatal serum ferritin concentration was evaluated only at one time in the whole pregnancy, can over or underestimate its association with birth weight as the exposure status to the deficiency might not be fixed. As birth weight is known to decline by 5–7% in the first three days of life [64], the study might have underestimated the birth weight with an equivalent fraction. Because of the community-based nature of our study, it was only possible to weigh newborns within 72 h of birth. The observational study nature of the data limits the possibility of identifying observed relationships. Due to the above reasons, the investigators recommend longitudinal national studies to assess the association between prenatal iron status and LBW.

## Conclusion

We found that low birth weight is of public health significance in this predominantly rural setting and demands attention in intervention in the community. Iron deficiency during pregnancy is found to harm birthweight. There is a risk of low birth weight in babies born to iron deficient mothers. Iron and folic acid supplementation, maternal under-nutrition, stunting, and sex of neonates were identified as predictors of low weight at birth. The study witnessed that there is an increased risk of low birth weight in neonates born to iron-deficient mothers. Health interventions must address targeted strategies promoting desirable food behavior and nutritional practices. These include; promoting the consumption of diversified and rich iron food to improve the maternal nutritional status. A continued effort is needed in enhancing universal access and compliance with IFA supplementation to improve maternal health. The reduction of low birth weight requires a comprehensive strategy that is based on a life-cycle approach to the problem. Thus, planners should consider the whole reproductive cycle and create a combination of strategies that are complementary and comprehensive across the vulnerable periods for women during pregnancy and their infants.

## Data Availability

The minimal data set used to reach the conclusions is available within the manuscript. Moreover, additional data required can be obtained from the corresponding author on a reasonable request.

## Abbreviations

ANC: Antenatal Care
ASF: Animal Source Food
DDS: Dietary Diversity Score
EDHS: Ethiopian Demographic and Health Survey
IDA: Iron Deficiency Anemia
FVS: Food Variety Score
HDS-HRC: Health Demographic Surveillance and Health Research Center
PCA: Principal Component Analysis
WHO: World Health Organization and Health Research Center
LBW: Low Birth weight
PCA: Principal Component Analysis
WHO: World Health Organization
